# Identification of key genes and signaling pathways of liver cancer and model construction for prognosis and diagnosis based on bioinformatics analysis

**DOI:** 10.1371/journal.pone.0325610

**Published:** 2025-06-04

**Authors:** Benzun Wei, Yao Zheng, Shuaijun Yu, Aiyun Wang, Xiao Lyu

**Affiliations:** 1 Department of Hepatobiliary Surgery, Zibo Central Hospital, Zibo, Shandong, China; 2 Department of Pathology, Zibo Central Hospital, Zibo, Shandong, China; 3 Department of Critical Care Medicine, Huantai County Hospital of Traditional Chinese Medicine, Zibo, Shandong, China; 4 Department of Pathology, Zibo Municipal Hospital, Zibo, Shandong, China; 5 Department of Medical Oncology, Zibo Central Hospital, Zibo, Shandong, China; Tulane University School of Medicine, UNITED STATES OF AMERICA

## Abstract

**Objective:**

This study aims to identify key genes, biomarkers, and associated signaling pathways involved in liver cancer progression by analyzing differentially expressed genes (DEGs) between normal and cancerous liver tissues, with the goal of establishing diagnostic and prognostic models for liver cancer.

**Methods:**

Two datasets, GSE39791 and GSE84402 from GEO, and clinical data from TCGA were selected. Differentially expressed genes (DEGs) were identified using the “limma” package in R, and volcano plots were generated. Functional enrichment of DEGs was performed with Gene Ontology (GO) and Kyoto Encyclopedia of Genes and Genomes (KEGG) analysis. Logistic regression and multivariate Cox regression models were established for diagnostic and prognostic prediction. The immortalized liver cell line THLE-3 and HepG2 cells were used to verify key gene expression via RT-qPCR and Western blot. HepG2 cells were transfected to up- and down-regulate SNAPC2 expression, and cell proliferation, migration, and apoptosis were assessed using CCK-8, colony formation, scratch, transwell migration assays, and flow cytometry with Annexin V-PE/7-AAD staining. Additionally, Gene Set Enrichment Analysis (GSEA) of SNAPC2 revealed its involvement in cancer-related pathways.

**Results:**

Bioinformatics analysis identified 10,961 down-regulated and 3,321 up-regulated genes in the GSE39791 and GSE84402 datasets, and 272 down-regulated and 4,855 up-regulated genes in TCGA data. GO and KEGG analysis revealed 3,820 co-DEGs associated with processes like cell differentiation and morphogenesis. CDCA8, GRPEL2, HAVCR1, MT3, MYCN, NDRG1, PHOSPHO2, SNAPC2, SOCS2, and TXNRD1 were selected to construct prognostic models, and MYCN, NDRG1, TXNRD1, SNAPC2, PHOSPHO2, and CDCA8 for diagnostic models. Western blot validation showed upregulation of CDCA8, GRPEL2, HAVCR1, MYCN, NDRG1, PHOSPHO2, SNAPC2, and TXNRD1 in liver cancer tissues, correlating with poor prognosis. Moreover, reduced SNAPC2 expression in HepG2 cells led to decreased proliferation and migration, and increased apoptosis, suggesting SNAPC2 plays a role in liver cancer progression by promoting cell proliferation and migration.

**Conclusion:**

CDCA8, GRPEL2, HAVCR1, MT3, MYCN, NDRG1, PHOSPHO2, SNAPC2, SOCS2, TXNRD1 were key genes for liver cancer prognosis and diagnosis. Moreover, lowering SNAPC2 expression could improve the prognosis of liver cancer through decreasing proliferation and migration s and increasing apoptosis of cancer cell.

## Introduction

Liver cancer is a malignant tumor that occurs in the liver, including primary liver cancer and metastatic liver cancer. Primary carcinoma of liver is a kind of cancer that occurs in liver cells or intrahepatic bile duct cells. It is the fourth common malignant tumor and the third cause of tumor death in the world, which seriously threatens the life and health of people [1]. Primary liver cancer mainly includes three different pathological types: hepatocellular carcinoma (HCC), intrahepatic bile duct (ICC) and HCC-ICC mixed type [2]. There are great differences in pathogenesis, biological behavior, histological morphology, treatment methods and prognosis among these three types of liver cancer. Among them, hepatocellular carcinoma accounts for more than 85% −90%, and has high degree of malignancy, strong infiltration and metastasis. In February 2019, Harvard Medical School and other research institutions included a follow-up study of about 120,000 subjects [3]. It was found that eating more whole grains was associated with a lower risk of hepatocellular carcinoma. The risk of hepatocellular carcinoma was significantly reduced by 37% compared with the population who ate the least.

The long-term efficacy in liver cancer depends on early diagnosis and treatment, for example, the combination of alpha-fetoprotein and imaging examination is the main auxiliary means of early diagnosis [4]. The treatment methods of liver cancer include hepatectomy, liver transplantation, local ablation, TACE, radiotherapy and other means [5]. For primary liver cancer, some patients with mild disease have a better prognosis. The prognosis of secondary liver cancer is related to the nature of primary cancer, the time of liver metastasis, the severity of primary and metastatic cancer at the time of discovery, the sensitivity of tumor to drug treatment, and individual factors. Data showed that about 70% of HCC recurred within 5 years after resection or ablation, and 75% of HCC recurred within 2 years after liver transplantation [6]. Therefore, screening markers related to early diagnosis, invasion and metastasis of HCC is of great significance to improve the therapeutic effect and prognosis of HCC.

The present study provides targets of genes or pathways for therapeutic as well as diagnostic strategies in the future. Moreover, this study firstly identified and verified the effect as well as underlying mechanisms of SNAPC2 on improving liver cancer prognosis, enhancing the understanding of SNAPC2 for liver cancer prognosis.

## Materials and methods

### Data sources

The liver cancer-related gene microarray data set in this study was obtained from Gene Expression Omnibus (GEO). The “liver cancer” was searched by GEO database (https://www.ncbi.nlm.nih.gov/geo/) to obtain GSE39791 and GSE84402 data sets for subsequent research. GSE39791 was based on GPL10558 platform, this gene expression profile was generated from tumor and matched non-tumor surrounding tissues of 72 hepatocellular carcinoma (HCC) patients. GSE84402 was based on GPL570 platform, including 14 pairs HCC tissues and corresponding non-cancerous tissues.

For further evaluating the effects of selected genes on clinic, the gene expression profile and corresponding clinical data were downloaded from TCGA (https://portal.gdc.cancer.gov/) by checking “liver and intrahepatic bile ducts”, “TCGA”, “TCGA-LIHC” for “cases” and “transcriptome profiling”, “Gene Expression Quantification” for “files”. In this data set, valid expressing data of 50 normal samples and 374 tumor samples and clinical information of 369 tumor samples could be utilized for the analysis of this study. The expression profile of this data in TCGA represented the normalized count of each gene in every sample.

### Data processing and identification of differential genes

Since there were two datasets from GEO, which were based on different platform, different samples, different groups and different experimenters, integrating all samples from two datasets above and utilizing “sva” package in the R computing environment for batch normalization to avoid potential variables and heterogeneity, improving the reliability of results.

Performing the differential analysis separately on data from GEO and TCGA by comparing normal tissues and tumor tissues utilizing “limma” package in the R computing environment. Genes would be screened as being differentially expressed when |log2FC| > 1 and P value < 0.05. The “plot” function was utilized for volcano map. Finally, using “VennDiagram” package to get intersection of genes which are both differentially expressed in GEO and TCGA databases.

### Identification of signaling pathway

Functional enrichment analysis was performed by mapping all DEGs to terms in the GO database. The analysis of GO term was classified into three subgroups: biological process (BP), cellular component (CC) and molecular function (MF). Moreover, all DEGs were also mapped to the KEGG database. The P-value < 0.05 and q-value < 0.05 were set as statistical threshold for searching for significantly enriched pathways. All these analyses were down in the R computing environment and mainly by the packages of “clusterProfilier” and “org.Hs.e.g.,db”. The bar plots, bubble plots and a network of relations between pathways were plotted to describe the enrichment of functions.

### Friend analysis and correlation analysis

Friend analysis measures the functional similarity between genes by calculating their semantic similarity in the MF, CC, and BP ontologies, and taking the geometric mean of these values. This analysis utilizes the mgeneSim function from the R package ‘GOSemSim’, combined with the Wang method to compute semantic similarity, ultimately generating a functional similarity matrix between genes.

Correlation analysis was performed on 10 genes separately in TCGA data and GEO data. The ‘cor’ function was used to calculate the correlation matrices of gene expression data, and the ‘ggcorrplot’ package was utilized to generate correlation heatmaps, providing a visual representation of the relationships between genes.

### Establishment and verification of prognostic model

Merging the profile of expression and survival data from TCGA, and performing univariate Cox regression analyze using the intersecting genes by the “survival” package in the R computing environment. A P-value < 0.05 was used for selecting genes for LASSO which is aiming at avoiding over-fitting and deleting genes which are tightly correlated.

Constructing a prognostic signature for patients of liver cancer by processing DEGs acquired after LASSO with the multivariate Cox regression analyze. A “step analyse” was performed to further excluding genes which are not closely associated with prognosis and the cut-off point was determined by the “survminer” package. There is a risk score acquired by this prognostic formula:


$$RiskScore=\sum_{i=1}^{n}Coef_{i}*x_{i}$$

In which for each gene in this signature, $Coef_{i}$ is their coefficients and $x_{i}$ is their normalized count. For verifying the ability of this diagnostic model, differentiating subgroups of high and low gene expression levels as well as high and low risk score and Kaplan-Meier survival curves were plotted to estimate the cumulative probability of surviving in a given time duration for patients in different subgroups. Further, the efficiency of the prognostic model was verified using operating characteristic curve (ROC) curve at 1-, 3-, 5-, and 8- years.

### Establishment and verification of diagnostic model

Based on the merged and normalized GEO data sets, acquiring the expression data of those 10 genes in prognostic model in both tumor samples and normal samples. To avoid the effect of over-dispersion, quasi-binomial logistic regression was used to construct the diagnostic model instead of binomial logistic regression. Finally, diagnostic model was constructed by 6 genes of MYCN, NDRG1, TXNRD1, SNAPC2, PHOSPHO2 and CDCA8 using logistic regression. Moreover, the Concordance index (C-index) was calculated and the time-dependent receiver operating characteristic curve (ROC curve) with the area under it (time-dependent AUC) was plot were to effectively evaluate the discriminative ability of the diagnostic model, a nomogram based on the 6 genes was built for subsequent clinical use and a calibration plot was used to determine the calibrating ability.

### GSEA analysis

For GSEA, SNAPC2 expression was treated as a numeric variable. The Pearson correlation coefficient of other genes and SNAPC2 expression was calculated, and then the genes were sequenced according to the correlation coefficient. Using the hallmark gene sets deposited in the GSEA Molecular Signatures Database resource (h.all.v7.1.symbols.gmt).

### Cell culture

The THLE-3 and HepG2 were purchased from the American Type Culture Collection (ATCC) (Beijing Zhongyuan Polymer Biotechnology Co., Ltd., Beijing, China) and were cultured in the Dulbecco’s Modified Eagles Medium (DMEM) (PM150210, Wuhan Punosa Life Technology Co., Ltd., Wuhan, China) supplemented with 10% fetal bovine serum (FBS) (164210, Wuhan Punosa Life Technology Co., Ltd., Wuhan, China) and 1% penicillin as well as 1% streptomycin (15140122, Thermo Fisher Scientific, Shanghai, China). We confirmed that mycoplasma testing has been done for the cell lines used in this study and the short tandem repeats (STR) identification showed that there was no cross contamination in the cell lines used in this study.

### Gene knockdown and overexpression

Lentivirus containing vectors targeting SNAPC2 knockdown (sh-SNAPC2) as well as SNAPC2 overexpression (OE-SNAPC2) and vector targeting none (NC) were produced by Shanghai Genechem Co., Ltd. (Shanghai, China). HepG2 cells were transfected by lentiviral particles for 3 days according to the manufacturers’ instructions. Since a puromycin resistance gene was also expressed by the vectors, puromycin (2.5 μg/mL) was utilized for screening transfected cells. After selection, choosing cells lines showing optimal gene knockdown and overexpression for subsequent experiments.

### CCK-8 assay and colony-forming assay

For performing the CCK-8 assay, transfected cells were seeded in 96-well plates, added with 100 μL CCK-8 solution and cultured at 37 °C for 4 h. measuring the absorbance at 450 nm with a microplate reader (Multiskan Sky, Thermo Fisher Scientific, Shanghai, China). In addition, for colony-forming assay, transfected cells were firstly left untreated for 48h. Then, cells were trypsinized and dispensed into tissue culture dishes with a density of 1000 counts/wells. Culturing cells for 14 days before they were fixed with 1 mL 4% paraformaldehyde and stained with Giemsa for visualization.

### Cell scratch test and transwell migration assay

Firstly, two horizontal lines were drawn on the back side of the 12 wells, and about 1.5 × 10^5^ cells were added to every well according to designed groups. The next day, changing into medium with low concentration and using a sterile pipette tip of 10 μL to make a single scratch vertical to the pre-drawn lines. Washing cells for three times, followed by culturing cells in the medium with 0.5% FBS in it and incubated in 5% CO_2_ at 37 °C. Taking photos for scratch at 0, 24 and 48 h (CKX53, Olympus, Tokyo, Japan), the migration ratio was calculated according to the scratch images through ImageJ (National Institutes of Health). Moreover, another assay for measuring migration was performed using the transwell kit (3422, Corning, New York, USA). Cell suspension (100 μL) of 1 × 10^5^ cells with no serum in medium were added into the upper chamber and complete medium (600 μL) with 30% FBS in it was added to the lower chamber. After being incubated for 24 h at 37 °C, removing the nonmetastatic cells which were on the membrane top surface and stained the metastatic cells which were on the membrane lower surface with Giemsa. Subsequently, metastatic cells were examined under an inverted fluorescent microscope (CKX53, Olympus, Tokyo, Japan) at 100 × magnification. Three random fields were photographed for counting cells and the average number of migrated cells was used as a quantification of migration capacity.

### Apoptosis analysis

Transfected cells were cultured for 24 h and harvested by trypsinization with no EDTA. After being washed twice with cold PBS and being collected following centrifugation, cells were re-suspended in the 1 × binding buffer (1 × 10^6^ cells/mL). Subsequently, 100 μL cell suspension with 1 × 10^5^ cells in it were stained with 5 μL Annexin-V-PE and 5 μL 7-AAD (Annexin V-PE/7-AAD apoptosis kit, MA0429, Meilunebio, Dalian, China) at a room temperature for 15 min. The operations of staining were fulfilled in dark condition and cell apoptosis was measured by flow cytometer (NovoCyte, Agilent Technologies Co., Ltd., Beijing, China). Results were analyzed by CellQuest and visualized by two-color dot plot.

### RT-qPCR

Extracting total RNAs from cells/tissues by TRIzol^®^ (CW0580S, Jiangsu Cowin Biotech Co., Ltd., Jiangsu, China) and evaluating RNA purity (OD260 nm/OD280 nm = 1.8–2.2) by a full-wavelength spectrophotometer (mμLtiskan sky, Thermo Fisher Scientific, Shanghai, China). According to the manufacturers’ instruction, reversely transcribing RNAs to cDNAs using a reverse transcription kit (AG11706, Accurate Biotechnology (Hunan) Co., Ltd., Hunan, China). The fluorescence quantitative instrument for PCR (CFX connect, Bio-Rad Laboratories (Shanghai) Co., Ltd., Shanghai, China) was utilized for performing qPCR following a cycling program of thermal. Relative levels of expression were normalized to the geometric mean of expression of the housekeeping gene GAPDH and 2^-ΔΔ^^Cq^ method was used for processing the data.

### Western blot

Extracting proteins from tissues or cells and collecting the supernatant with total protein in it. Total protein concentrations of the whole tissue- or cell-extracts were measured using BCA Protein Quantitation kit (P0012, Beyotime Biotechnology Co., Ltd., Shanghai, China). Adding the loading buffer at 1/4 of the total protein solution volume, then cell lysis was denatured at 100 °C spoiling water for 15 min for the subsequent SDS-PAGE electrophoresis. Subsequently, proteins were transferred to polyvinylidene fluoride (PVDF) membranes (IPVH00010, Millipore, Billerica, Massachusetts, USA) which were blocked with 5% skim milk (G5002, Serveicebio, Wuhan, China) at room temperature for 1 h, and were incubated with primary antibodies (CDCA8: 1:1000 dilution, Cat. No. A15463, Abclonal; GRPEL2: 1:1000 dilution, Cat. No. A8339, Abclonal; HAVCR1: 1:1000 dilution, Cat. No. A2831, Abclonal; MT3: 1:1000 dilution, Cat. No. Sc-293488, Santa Cruz; MYCN: 1:3000 dilution, Cat. No. A22175, Abclonal; NDRG1: 1:1000 dilution, Cat. No. A2142, Abclonal; PHOSPHO2: 1:1000 dilution, Cat. No. Sc-398826, Santa Cruz; SNAPC2: 1:1000 dilution, Cat. No. A16069, Abclonal; TXNRD1: 1:1000 dilution, Cat. No. A4725, Abclonal; SOCS2: 1:1000 dilution, Cat. No. A9190, Abclonal; GAPDH: 1:50000 dilution, Cat. No. 60004–1-Ig, Proteintech) at 4 °C overnight. Washing the membranes with PBST (PBS, 0.05% Tween-20) for 15 min and incubating membranes with the secondary antibody (horseradish peroxidase labelled goat anti-mouse IgG: 1:5000 dilution, Cat. No. ZB-2305, ZSGB-Bio; horseradish peroxidase labelled goat anti-rabbit IgG: 1:5000 dilution, Cat. No. ZB-2301, ZSGB-Bio) at room temperature for 40 min. After washing membranes with PBST for 15 min, the antibody-reactive bands were revealed (Tanon-5200, Shanghai Tanon Life Science Co., Ltd., Shanghai, China). Here, GAPDH was used as the internal reference.

### Statistical analysis

Experimental data from experiments conducted repeatedly were presented as the mean ± standard deviation. The data and images of all experiments were analyzed through GraphPad Prism 8.0.2 pairwise and statistical analyses between two groups or multi-group comparisons were performed using the t-test or analysis of variance. A P value < 0.05 was considered to indicate a statistically significant difference.

## Results

### Identification of DEGs

**Fig 1:**
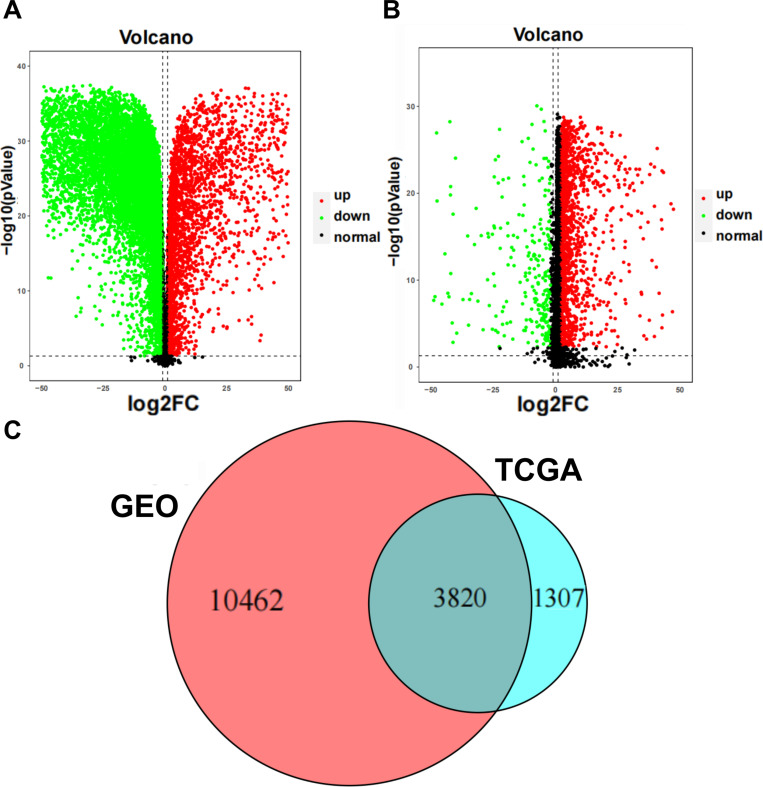
Identification of DEGs. **(A)** Volcano plots of DEGs in GEO. **(B)**Volcano plots of DEGs in TCGA. **(C)** The Venn diagram showed 3820 overlapping DEGs.

This study compared the gene expression of GSE39791 and GSE84402 from the GEO database and TCGA database in tumor tissues and non-tumor tissues of patients with liver cancer. Based on the merged and normalized data sets of GSE39791 and GSE84402, 10961 down-regulated genes and 3321 up-regulated genes were identified. TCGA data set included 272 down-regulated genes and 4855 up-regulated genes. The description of DEGs in GEO database and TCGA database were shown in Fig 1A and Fig 1B, the Fig 1C indicated that there were 3820 DEGs differentially expressed in both GEO database and TCGA database. Among these 3820 intersecting DEGs, 208 DEGs were down-regulated and 3612 DEGs were up-regulated. The gene names of the 3,820 overlapping DEGs have been included in S1 File.

### GO annotation analyses of DEGs

**Fig 2:**
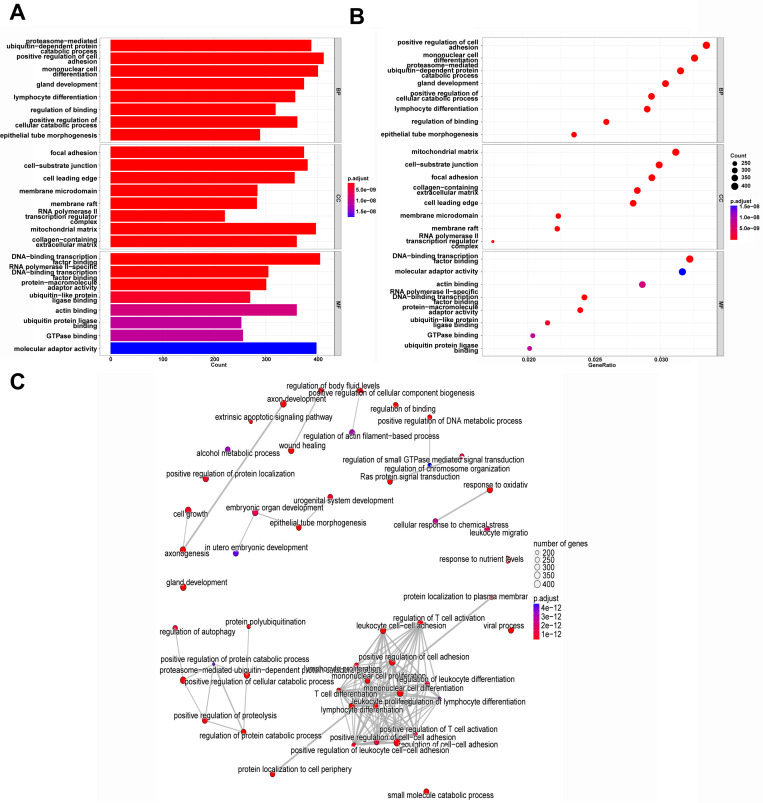
GO annotation analyses of DEGs. **(A)** The bar plot of GO annotation analyses of top 8 enriched pathways in BP, CC and MF. **(B)** The bubble plot of GO annotation analyses of top 8 enriched pathways in BP, CC and MF. **(C)** The network of interaction between top 50 enriched pathways.

The bar plot and bubble plot of GO analysis (Figs 2A and 2B) showed the DEGs were associated with catabolic process, adhesion and binding, cell differentiation and morphogenesis for Biological Process (BP), focal adhesion, cell-substrate junction, cell leading edge, membrane raft, regulator complex for RNA polymerase II transcription and matrix for Cell Component (CC), adaptor activity and binding for Molecular Function (MF). Fig 2C showed the top 50 pathways enriched and the relations among them. The pathways for life cycle of T cell as well as differentiation, adhesion and activation of other cells were relevant to each other and were largely enriched by the DEGs. Moreover, cellular catabolic process was relevant to the protein catabolic process and autophagy was relevant to catabolic process.

### KEGG pathway enrichment analyses of DEGs

**Fig 3:**
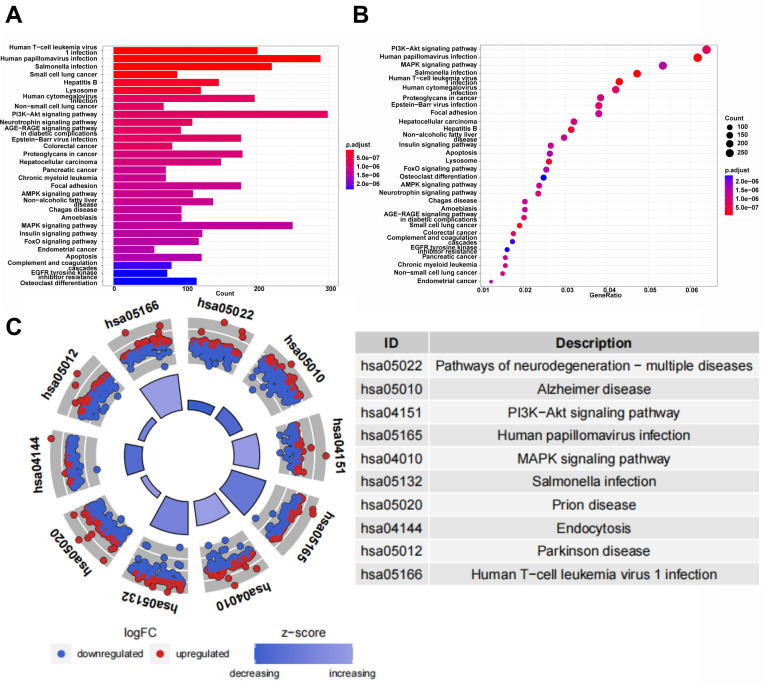
KEGG pathway enrichment analyses of DEGs. **(A)** The bar plot of top 30 enriched KEGG pathways. **(B)** The bubble plot of top 30 enriched KEGG pathways. **(C)** The circle plot and the table of description for the top 10 enriched KEGG pathways.

The results of KEGG pathway analysis (Figs 3A, 3B and 3C) suggested that the DEGs were mainly related to several types of cancers, virus infection, life cycle of cells and signaling pathways such as PI3K-Akt signaling pathway, MAPK signaling pathway, Insulin signaling pathway, AMPK signaling pathway, Neurotrophin signaling pathway and AGE-RAGE signaling pathway.

### Construction and verification of prognostic model

**Fig 4:**
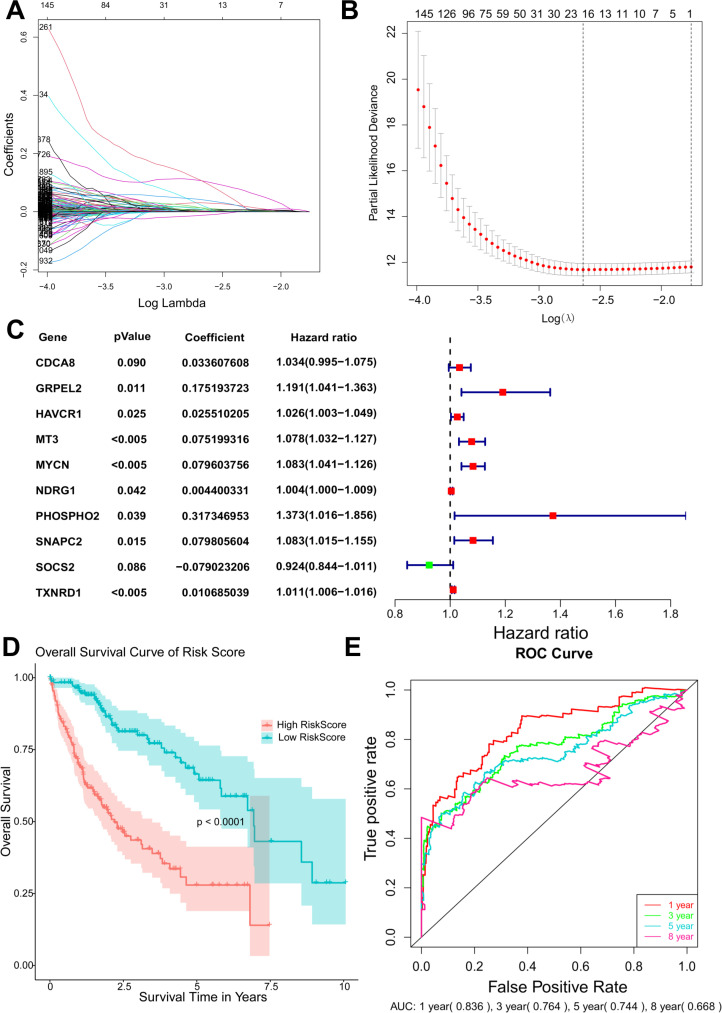
Construction and verification of prognostic model. **(A)** Coefficient profile of LASSO. **(B)** The selection of tuning parameter (lambda) based on the minimum criteria for overall survival and by 10-fold cross-validation. **(C)** The forest plot for summarizing the prognostic model, showing the coefficients, hazard ration (including the range) and the p value of every ten selected genes. **(D)** The Kaplan-Meier survival curve of patients in high-risk and low-risk groups. **(E)** The ROC curves of the model estimating 1-, 3-, 5- and 8-year overall survival.

The univariate Cox regression analyze was performed on the overlapping DEGs and according to the statistical threshold of P-value < 0.05, 2141 genes were screen as being closely relevant to the prognosis and were further analyzed by LASSO regression. 16 DEGs were screened for subsequent analysis by LASSO regression (Figs 4A and 4B) and by univariate Cox regression analysis, 10 most relevant genes: CDCA8, GRPEL2, HAVCR1, MT3, MYCN, NDRG1, PHOSPHO2, SNAPC2, SOCS2 and TXNRD1, were identified and utilized for constructing the prognostic signature. Fig 4C summarizes the multivariable survival analysis based on the expression of 10 genes. The hazard ratio analysis indicated that higher expression levels of HAVCR1, MT3, MYCN, NDRG1, PHOSPHO2, SNAPC2, and TXNRD1 were associated with poorer prognosis, whereas higher expression levels of SOCS2 were associated with a more favorable prognosis.

**Fig 5:**
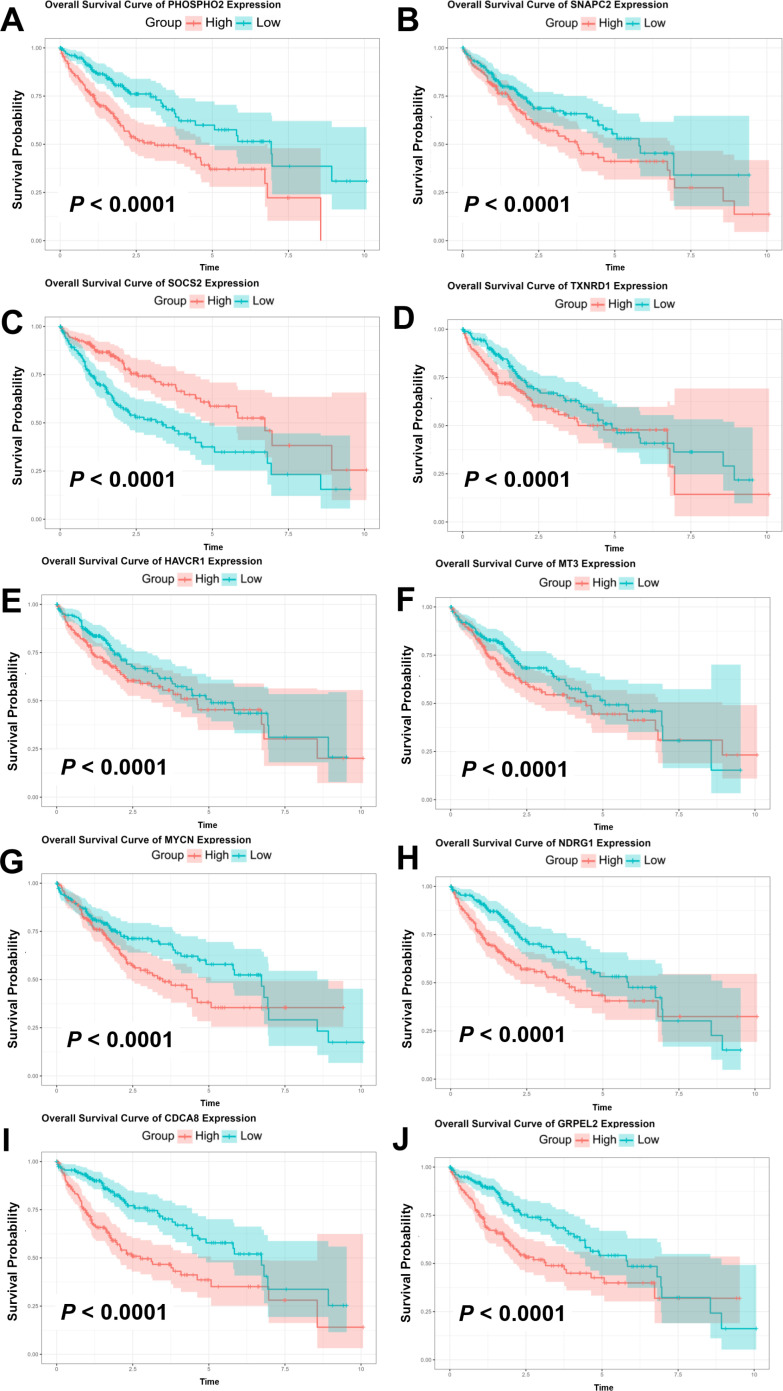
Kaplan-Meier survival curve. **(A-J)** The Kaplan-Meier survival curve of different genes in high-expression and low-expression groups.

According to the model we established and the risk score calculated by the model, individuals with liver cancer could be stratified into two subgroups with discrete overall survival by the median risk score the group of high-risk and the group of low-risk. The Kaplan-Meier analysis was shown in Fig 4D, illustrating those patients with high-risk scores possessed more remarkably reduced overall survival compared with patients with low-risk score. The P-value < 0.0001 verified the promising efficacy of the model to estimate prognosis. Additionally, the ROC curve in Fig 4E showed the true positive rate along the y axis versus its false positive rate along the x axis. According to curves and the AUCs of different survival duration, the estimation accuracy of this model was reasonable since the AUC of 1-, 3- and 5-year overall survival were 0.836, 0.764 and 0.744, respectively. Although the AUC of the curve estimate 8-year survival was 0.668, which was referable, the curve itself was excessively vibrating, decreasing the accuracy and reliability of this model. The gene expressions were classified into two subgroups according to the median of expression levels, and the Kaplan-Meier survival curves of each signature gene were draw to assess the prognostic values of them (Fig 5). According to these curves, CDCA8, GRPEL2, HAVCR1, MT3, MYCN, NDRG1, PHOSPHO2, SNAPC2, SOCS2 and TXNRD1 were relevant to the overall survival of liver cance.

### Establishment and verification of diagnostic model

**Fig 6:**
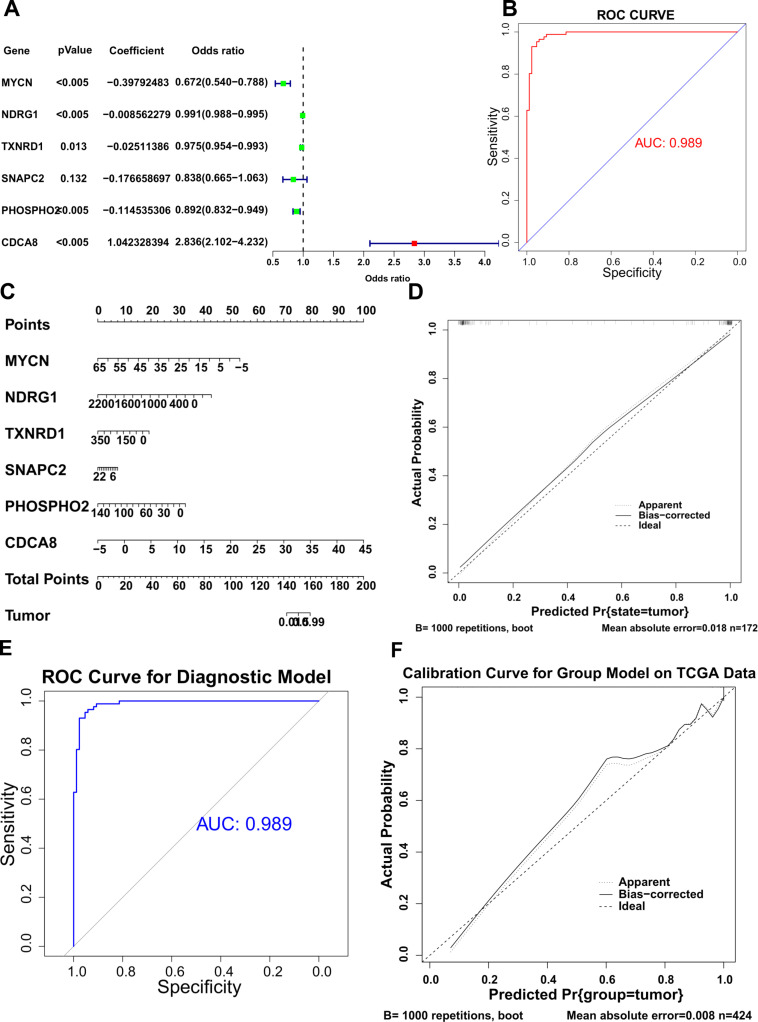
Establishment and verification of diagnostic model. **(A)** The forest of summarizing the diagnostic model. **(B)** The ROC curve of this diagnostic model. **(C)** The nomogram for the clinical utility of this model. **(D)** The calibration curves for the nomogram. **(E)** The ROC curve of this diagnostic model in TCGA data. **(F)** The calibration curves for the nomogram in TCGA data.

The P value acquired by the Pearson’s chi-squared test for the binomial and quasi-binomial logistic regression was 0.005, which was less than 0.05 and showed an over-dispersion. Therefore, the quasi-binomial logistic regression was used to construct the model for diagnosis. The result of model with 10 genes showed that 6 genes of MYCN, NDRG1, TXNRD1, SNAPC2, PHOSPHO2 and CDCA8 were more significantly associated with diagnosis of tumor according to the statistical filter of P < 0.05. The Pearson’s chi-squared test was then performed to examine the distinction of diagnostic efficacy between the 10-gene model and 6-gene model. The result of 0.0322 showed the distinction and therefore, the 6-gene model was more reliable as diagnostic signature. The summary of this model was shown in Fig 6A. The forest of summarizing the diagnostic model, showed the coefficients of every expressing level of genes. The P-value suggested the association of genes expression and the probability of experiencing liver cancer. The odds ration showed association between expression of genes and the presence of liver cancer: OR < 1 suggested individuals were more likely to experience liver cancer when the expression of the gene increases, and conversely, less expression of gene showed less probability of suffering from liver cancer. Therefore, an increasing expression of CDCA8 lead a higher probability of presenting liver cancer. The C-index was 0.989 and the AUC was 0.989 in the ROC curve (Fig 6B, the sensitivity along the y axis versus its 1-specificity along the x axis) described that along all range of specificity (or sensitivity), the sensitivity (or specificity) was reasonable, suggesting a reasonable estimation and discrimination accuracy of this model. In consequence, this model was capable of discrimination between individuals who experience liver cancer and individuals who do not. For clinical utility of this model, a nomogram was constructed incorporating these 6 genes (Fig 6C), a total point was calculated by the use of the expressing levels of 6 genes. The expressing level of each of these genes was given a point on the point scale axis, the total points could be easily acquired by adding each single point. Afterwards, estimating the probability of liver cancer by projecting the total point to the lower scale named “tumor”. Moreover, a calibration curve of the nomogram was presented in Fig 6D. The y axis represented the actual probability of liver cancer and x axis represented the predicted probability of liver cancer. The perfect prediction was corresponded to the ideal line. The Apparent line representing the entire cohort in our data (n = 172) and the Bias-corrected line representing bootstrapping (B = 1000 repetitions), indicated the performance of nomogram. In this plot, there were a close over-position between the apparent line and the Bias-corrected line and these two lines were closely near the ideal line, which illustrated that the predicted probabilities of experiencing liver cancer by the nomogram agreed well with the actual probabilities of experiencing liver cancer.

To validate the clinical prediction model, we further tested it on the TCGA liver cancer dataset. As shown in Fig 6E, the ROC curve of the model reached 0.985, demonstrating its excellent predictive performance. The calibration curve (Fig 6F) further confirmed the model’s high accuracy in predicting the phenotypes of TCGA liver cancer samples. These results indicate that the model exhibits strong generalization ability and reliable performance across different datasets, making it a powerful tool for clinical prediction in liver cancer.

### Validation of the level of key genes

**Fig 7:**
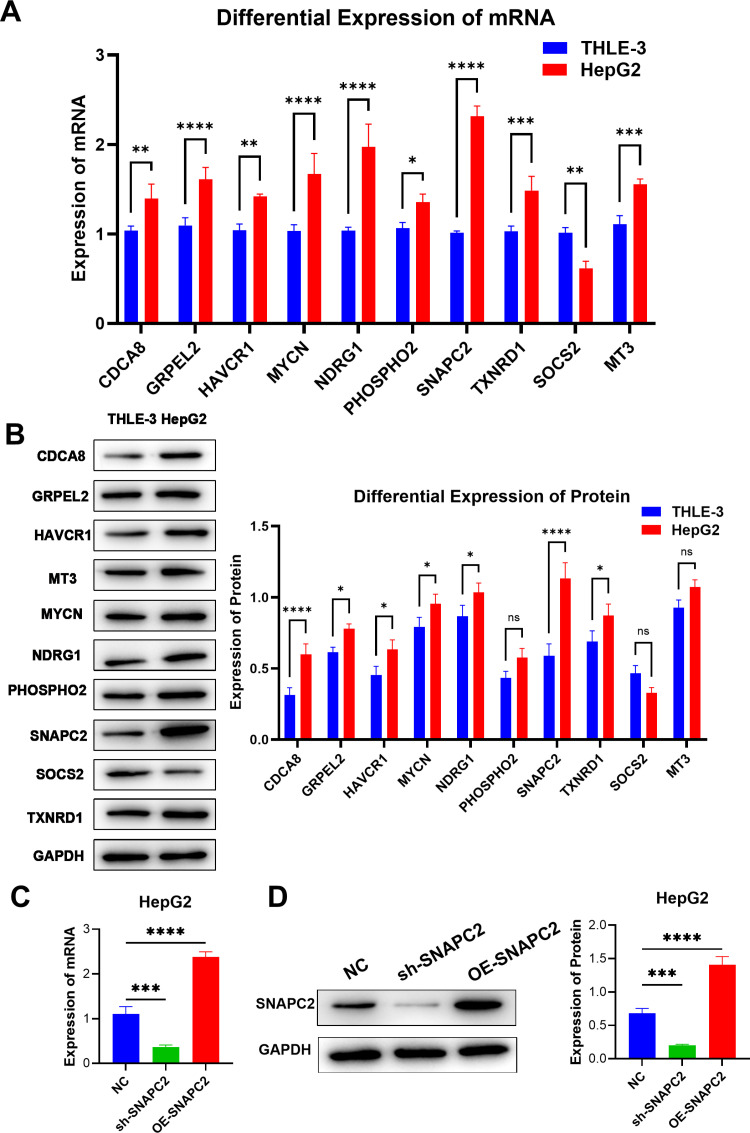
Validation of the level of key genes. **(A)** mRNA expressions of the 10 signature genes detected by RT-qPCR (n = 6). **(B)** Protein expressions of the 10 signature genes detected by Western Blot (n = 3). **(C)** SNAPC2 mRNA expression in transfected HepG2 cells detected by RT-qPCR (n = 6). **(D)** SNAPC2 protein expression in transfected HepG2 cells detected by Western Blot (n = 3). * P < 0.05, ** P < 0.01, *** P < 0.001.

In order to explore and verify the prognostic value of these signature genes, we determined their expression in cancer cells and normal cells by RT-qPCR (Fig 7A) and western blot (Fig 7B). The results showed that the expression of CDCA8, GRPEL2, HAVCR1, MYCN, NDRG1, PHOSPHO2, SNAPC2, MT3 and TXNRD1 was significantly up-regulated in liver cancer cells compared with normal cells. While the expression of SOCS2 was down-regulated. The results of RT-qPCR (Fig 7A) and Western Blot (Fig 7B) showed that the expression of SNAPC2 was down-regulated in HepG2 cells with SNAPC2 knockdown vector (si-SNAPC2) while up-regulated in HepG2 cells with SNAPC2 overexpression vector (OE-SNAPC2), which indicated that the transfection in the experiments was successful.

### SNAPC2 affected the proliferation, migration and apoptosis of liver cancer cells

**Fig 8:**
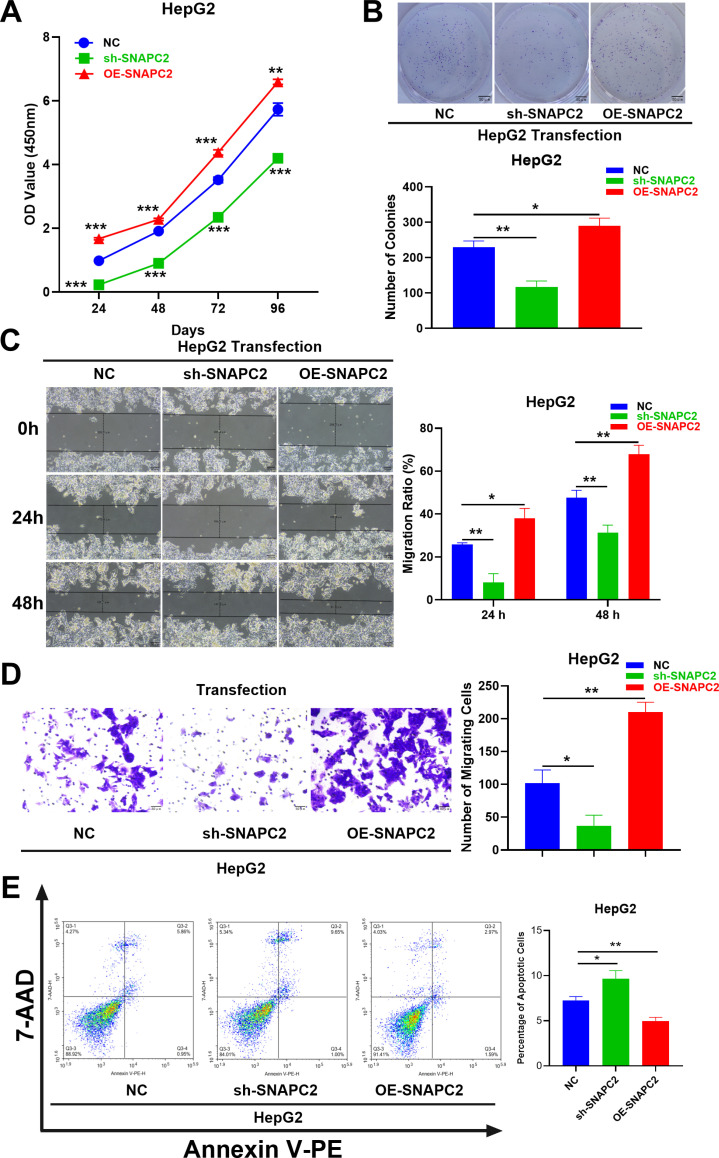
SNAPC2 affected the proliferation, migration and apoptosis of liver cancer cells. **(A)** The result of CCK-8 assays (n = 3). **(B)** The result of colony-forming assay (n = 3). **(C)** The result of cell scratch test (n = 3). **(D)** The result of transwell migration assay (n = 4). **(E)** The result of flow cytometry after transfected cells being double stained. * P < 0.05, ** P < 0.01, *** P < 0.001.

The result of CCK-8 assay was shown in Fig 8A, si-SNAPC2 cells possessed lower OD value while OE-SNAPC2 cells possessed higher OD value. The same result was shown in Fig 8B that si-SNAPC2 formed less colonies while OE-SNAPC2 formed more colonies. The results of CCK-8 assay and colony-forming assay indicated that the liver cancer cells with lower/higher expression of SNAPC2 possessed lower/higher level of proliferation. The results of cell scratch test (Fig 8C) and transwell migration assay (Fig 8D) showed that cells with lower expression of SNAPC2 migrated less than cells with higher expression of SNAPC2. The result of flow cytometry (Fig 8E) showed that the apoptosis of liver cancer cells with lower SNAPC2 expression was more than that of cells with higher SNAPC2 expression.

### SNAPC2 may act as a key transcription factor promoting the progression of liver cancer

**Fig 9:**
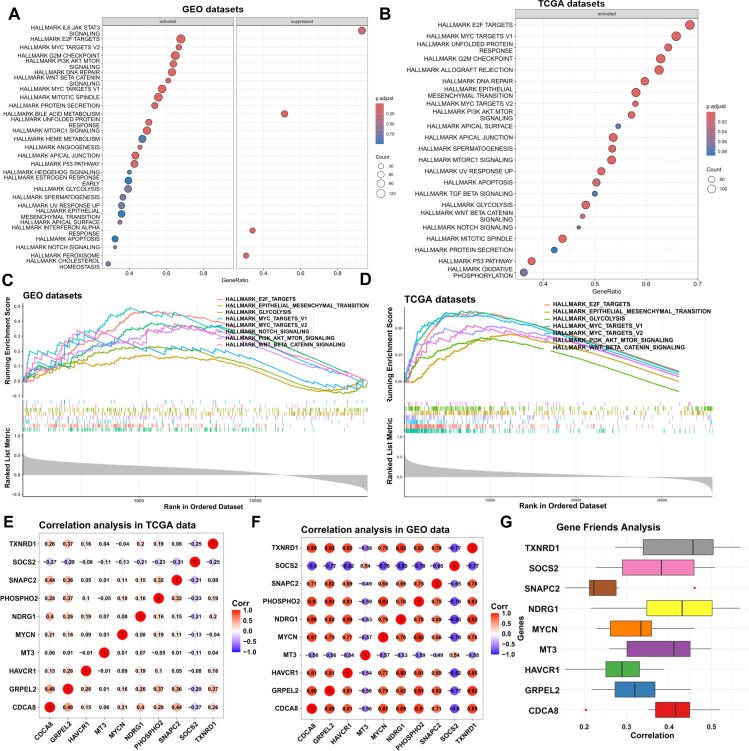
Exploring the mechanism of SNAPC2 in liver cancer. **(A)** GSEA enrichment analysis of single gene SNAPC2 in GEO data. **(B)** GSEA enrichment analysis of single gene SNAPC2 in TCGA data. **(C)** GSEA curves in GEO data. **(D)** GSEA curves in TCGA data. **(E-F)** Correlation analysis heatmap. **(G)** Boxplot display of Friend analysis results.

To further investigate the role of SNAPC2 in liver cancer, we performed single-gene GSEA (Gene Set Enrichment Analysis) on two datasets. As shown in Figs 9A-B, SNAPC2 activation is associated with several cancer-promoting pathways in liver cancer samples. Figs 9C-D demonstrate that SNAPC2 activates key pathways in both datasets, including “HALLMARK_MYC_TARGETS_V1”, “HALLMARK_E2F_TARGETS”, “HALLMARK_MYC_TARGETS_V2”, “HALLMARK_PI3K_AKT_MTOR_SIGNALING”, “HALLMARK_EPITHELIAL_MESENCHYMAL_TRANSITION”, “HALLMARK_GLYCOLYSIS”, “HALLMARK_WNT_BETA_CATENIN_SIGNALING”, and “HALLMARK_NOTCH_SIGNALING”. We hypothesize that SNAPC2, as a crucial transcription factor, may promote the transcription of key factors in these pathways, thereby activating downstream signaling and contributing to the progression of liver cancer.

Additionally, to investigate whether SNAPC2 exhibits coordinated interactions with other co-selected genes, we performed correlation analyses in both TCGA and GEO datasets. As shown in Fig 9E and Fig 9F, SNAPC2 demonstrated strong correlations with GRPEL2, CDCA8, and PHOSPHO2. However, the results of the Friend analysis in Fig 9G revealed that SNAPC2 exhibited weak functional similarity (in Molecular Function, Cellular Component, and Biological Process) with these genes, suggesting that SNAPC2 may not coordinate with them in the context of oncogenic mechanisms.

## Discussion

Patients with primary liver cancer lack typical clinical manifestations in the early stage. Once symptoms and signs appear, most patients with liver cancer have entered the middle and late stage. Therefore, the early diagnosis of liver cancer is of great significance for treatment. Bioinformatics screening and monitoring techniques are helpful for early diagnosis and intervention and good prognosis of patients with liver cancer [7]. In this study, 10961 down-regulated genes and 3321 up-regulated genes were identified from GEO data sets (merged and normalized by GSE39791 and GSE84402) and 272 down-regulated genes and 4855 up-regulated genes were identified from TCGA database. Based on these, 3820 differentially expressed genes in both GEO data sets and TCGA database, including 208 down-regulated genes and 3612 up-regulated genes were screened out. The referable prognostic signature was constructed by multivariate Cox regression analyze incorporating CDCA8, GRPEL2, HAVCR1, MT3, MYCN, NDRG1, PHOSPHO2, SNAPC2, SOCS2 and TXNRD1. The performance of this model was assessed by the Kaplan-Meier analysis and ROC curve. Additionally, a reasonable diagnostic signature was established by quasi-binomial logistic regression incorporating MYCN, NDRG1, TXNRD1, SNAPC2, PHOSPHO2 and CDCA8. The C-index and ROC curve were used to assess the estimation and a nomogram and a calibration curve were plot for further clinical utility.

Moreover, GO analysis and KEGG analysis of overlapping DEGs were carried out. The results of GO annotation showed that DEGs were associated with catabolic process, life cycle of cells as well as the adhesion, binding, differentiation and morphogenesis of cells [8–10]. The results of KEGG pathway analysis showed that the DEGs were mainly related to cancer and diseases, virus infection, life cycle of cells and several significant signaling pathways. Among those signature genes, the CDCA8 is significantly associated with the prognosis and has been verified could be an independent predictor for a poor prognosis in liver cancer [11]. Further, the gene set enrichment analysis (GSEA) between low and high CDCA8 expression datasets showed that CDCA8 was relevant to cell cycle, ErbB signaling pathway, MAPK signaling pathway, mTOR signaling pathway, Notch signaling pathway and p53 signaling pathway. In addition, the CDCA8 has been proved could promote the proliferation and invasion of pancreatic ductal adenocarcinoma by activating CD44 [12]. In this study, the Kaplan-Meier analysis of individual gene suggested that patients with high expression of CDCA8 possessed distinguished poor survival compare with patients with low expression of CDCA8. The gene of GRPEL2 was well verified in the maintaining of mitochondrial homeostasis which is meaningful to several biological process for cancer survival. It has been proved that the knockdown of GRPEL2 suppressed the growth, invasion and migration *in vitro*, as well as inhibited the growth of tumor *in vivo*. Moreover, the deficiency of GRPEL2 also lead cell apoptosis by increasing mitochondrial membrane potential (MMP) and accelerating reactive oxygen species (ROS) production [13]. HAVCR1 is an important role in renal regression and immunity and has been published could be an biomarker for the prognosis and diagnosis for pan-cancer [14] and also can be used for prognosis for gastric cancer [15]. MYCN is a member of an oncogene family named MYC and encodes a basic transcription factor N-MYC. The deregulation of the MYC oncogene family has been found in many types of cancer and is relevant to a poor prognosis [16]. The targeting of MYCN as a therapeutic approach has been considered broadly. Since it is “undruggable”, the focus is alternatively on the molecular mechanisms such as disrupting the transcription and translation of MYCN, decreasing the protein stability of N-MYC and its synthetic lethality [17]. The NDRG1, which is the abbreviation of N-MYC downregulated gene-1, is a target gene which is repressed by N-MYC [18]. This gene is a regulator of oncogenes and the function of it in cancer depends on the type of tumor cell and the status of cancer [19]. The overexpression of SOCS2 could inhibit NF-κB signaling pathway [20], TGFβ pathways [21] and JAK-STAT pathway [22]. The gene of TXNRD1 has been proved to be regulated by the Mrf2/keap1 pathway as an antioxidant [23] and closely correlated with mTOR signaling pathway [24]. Mtallothionein (MT) is a low molecular weight protein with metal binding ability and high induction property [25–27]. The structure of MT is highly conservative in biological evolution, and there are mainly four isomers. MT-Ⅰ and MT-Ⅱ are widely present in most mammalian visceral organs, especially liver and kidney cells, and participate in their function regulation. The distribution of MT-Ⅲ is mainly limited to the central nervous system, mainly distributed in astrocytes (concentrated in cell bodies and processes), following by neurons. It is also reported that a small amount of MT-Ⅲ is distributed in germ cells, small intestine, stomach, kidney and olfactory cortex cells [28].

However, among the 10 selected key genes in the present study, few studies were down for SNAPC2. Especially, there were no researches about SNAPC2 affecting liver cancer. In this present study, we firstly explored the effects of SNAPC2 on liver cancer. Based on the survival analysis on SNAPC2, we confirmed that the lower expression of SNAPC2 was related to the better prognosis of liver cancer. The results revealing proliferation, migration and apoptosis of transfected liver cancer cell HepG2 illustrated that lower the expression of SNAPC2 could decrease the proliferation and migration of liver cells while increase the apoptosis of liver cancer cells, thus improve the prognosis of liver cancer. Furthermore, as a transcription factor, SNAPC2 plays a crucial role in the mechanistic understanding of liver cancer. Our analysis indicates that SNAPC2 activates several key oncogenic pathways, including MYC, E2F, Notch, and PI3K signaling pathways, all of which are closely associated with tumor proliferation, metabolic reprogramming, and malignant transformation. Furthermore, SNAPC2 enhances glycolysis and epithelial-mesenchymal transition (EMT) in cancerous samples, which may promote tumor cell invasiveness and metastatic potential. Glycolysis, a hallmark of tumor cells, not only provides the necessary energy for tumor cell proliferation but also influences cancer progression by modulating the tumor microenvironment. EMT is a critical process by which tumor cells acquire invasive and metastatic properties. Through regulating these pathways, SNAPC2 may drive the progression of liver cancer. In summary, as a critical transcription factor, SNAPC2 not only provides novel insights into the mechanisms underlying liver cancer but also represents a promising therapeutic target for the treatment of this disease.

In the current research, one limitation of this study is the lack of further experimental validation of the downstream pathways regulated by SNAPC2. In future studies, we plan to further investigate the direct regulatory relationships of SNAPC2 with key genes in these pathways. Another limitation is that the in vitro experiments in this study used the HepG2 hepatoblastoma cell line. Although the results from these experiments validated our previous bioinformatics findings, this choice may impose certain limitations in further mechanistic exploration. Therefore, future studies will incorporate additional liver cancer cell lines with well-defined origins to further validate the reliability and generalizability of our findings and facilitate the exploration of subsequent mechanisms.

## Conclusion

CDCA8, GRPEL2, HAVCR1, MT3, MYCN, NDRG1, PHOSPHO2, SNAPC2, SOCS2 and TXNRD1 were verified associated with the prognosis of liver cancer and among those key genes, MYCN, NDRG1, TXNRD1, SNAPC2, PHOSPHO2 and CDCA8 were related to diagnosis of liver cancer. Moreover, the effect of SNAPC2 on improving prognosis were verified and demonstrated was through decreasing proliferation as well as migration of liver cancer cell and increasing apoptosis of liver cancer cell. The present study provides targets of genes or pathways for therapeutic as well as diagnostic strategies in the future and firstly reveals the effect as well as underlying mechanisms of SNAPC2 on improving liver cancer prognosis.

## Supporting information

S1 File The gene names of the 3820 overlapping DEGs. (DOCX)
