# Supplementary figures and images for: Identification of key genes and signaling pathways of liver cancer and model construction for prognosis and diagnosis based on bioinformatics analysis
Source: PLoS One. 2025 Jun 4;20(6):e0325610. doi: 10.1371/journal.pone.0325610 (PMC12136465; doi:10.1371/journal.pone.0325610)

7B/CDCA8


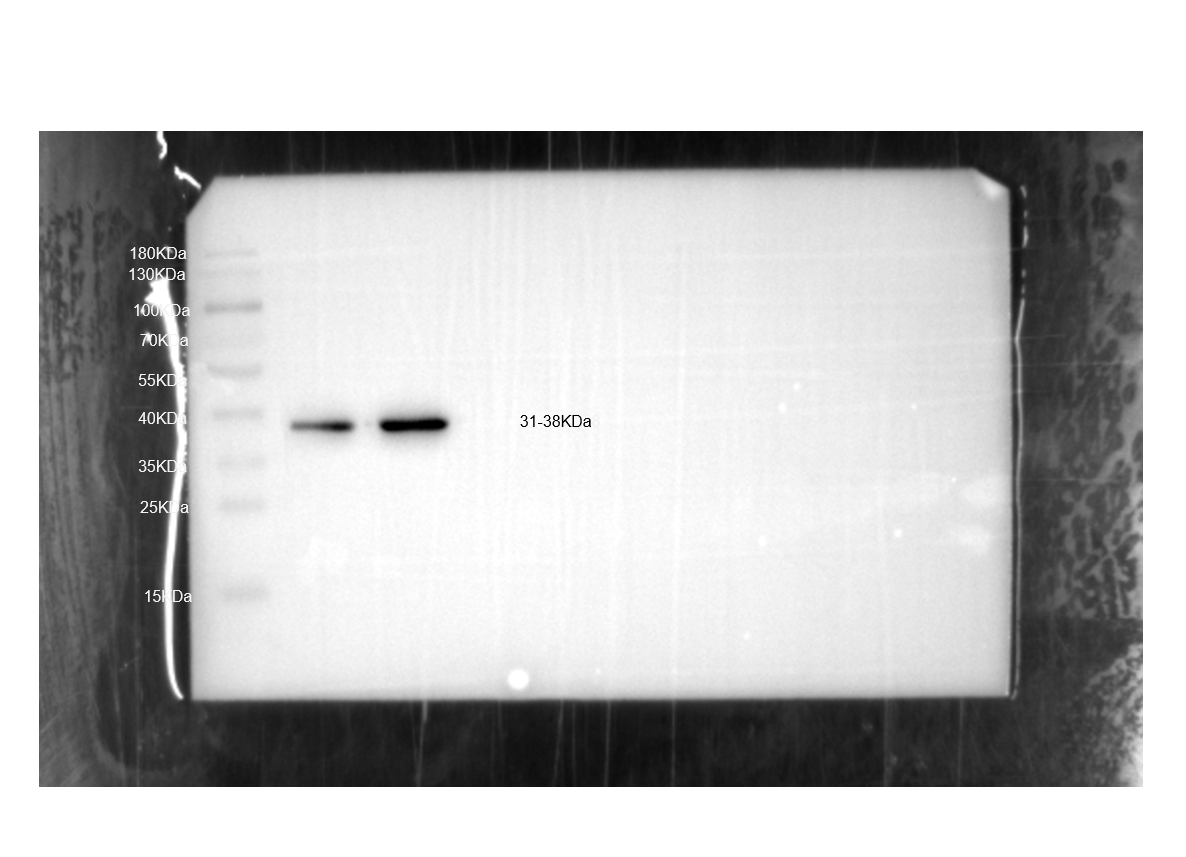


7B/GAPDH


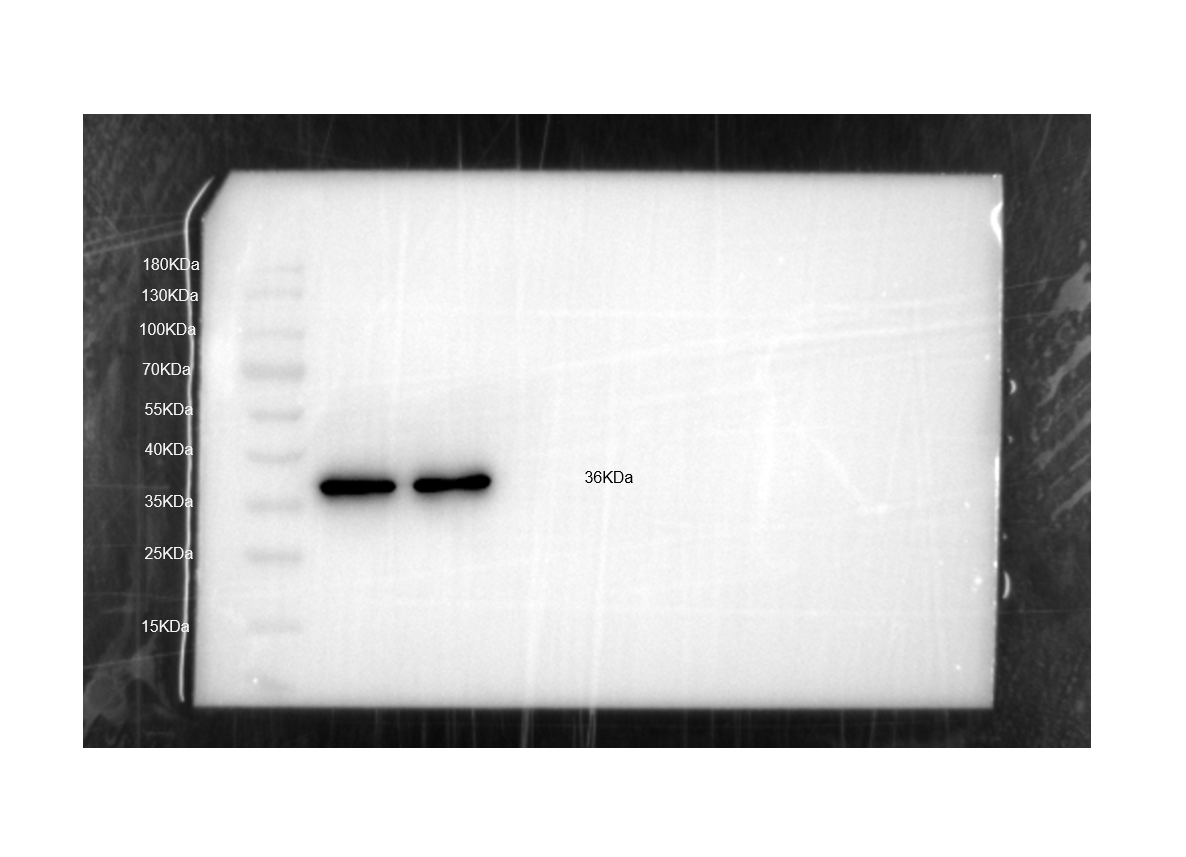


7B/GRPEL2


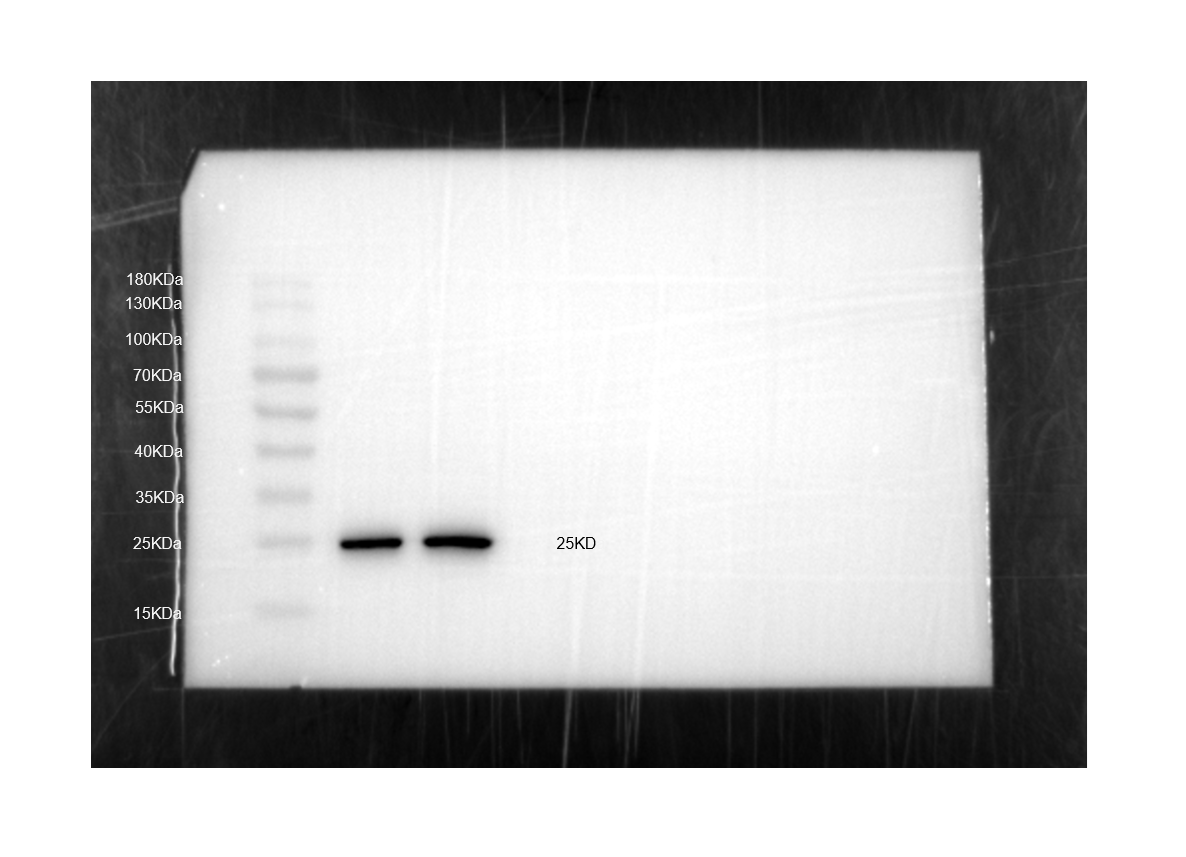


7B/HAVCR1


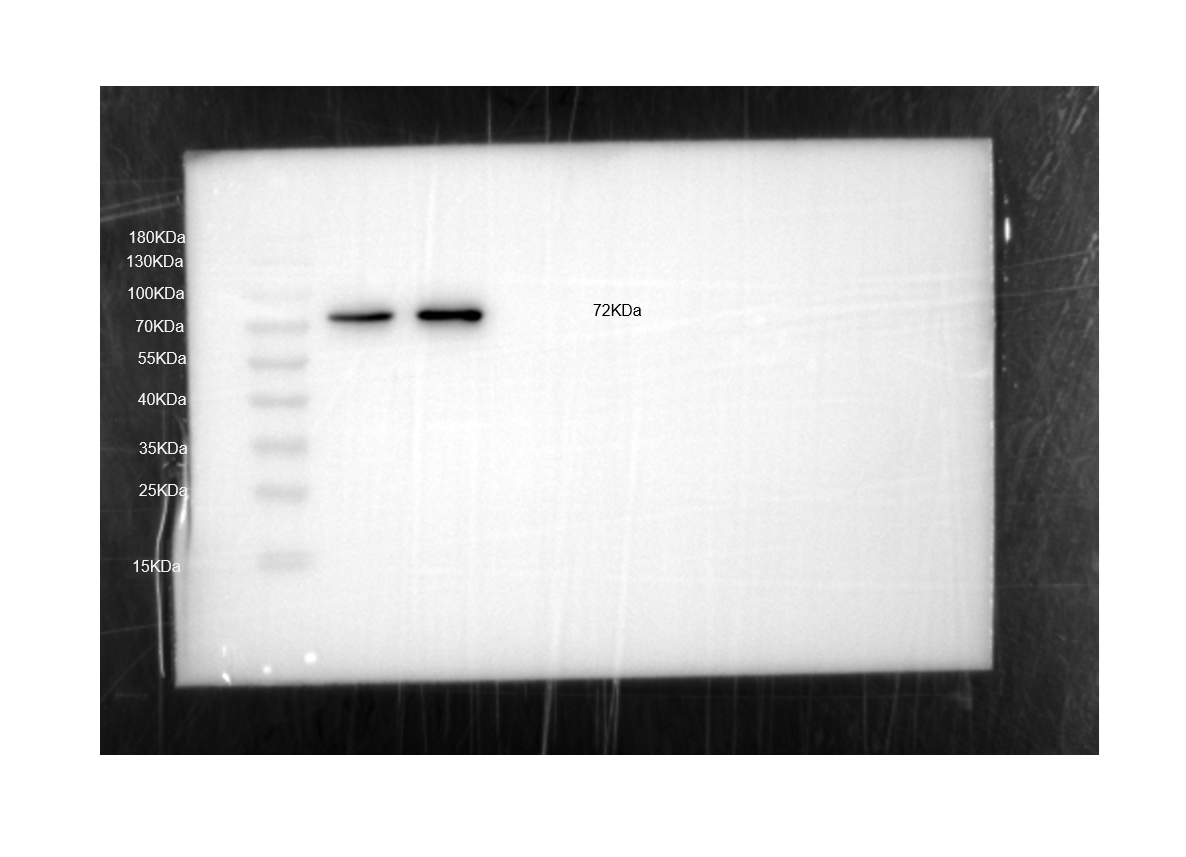


7B/MT3


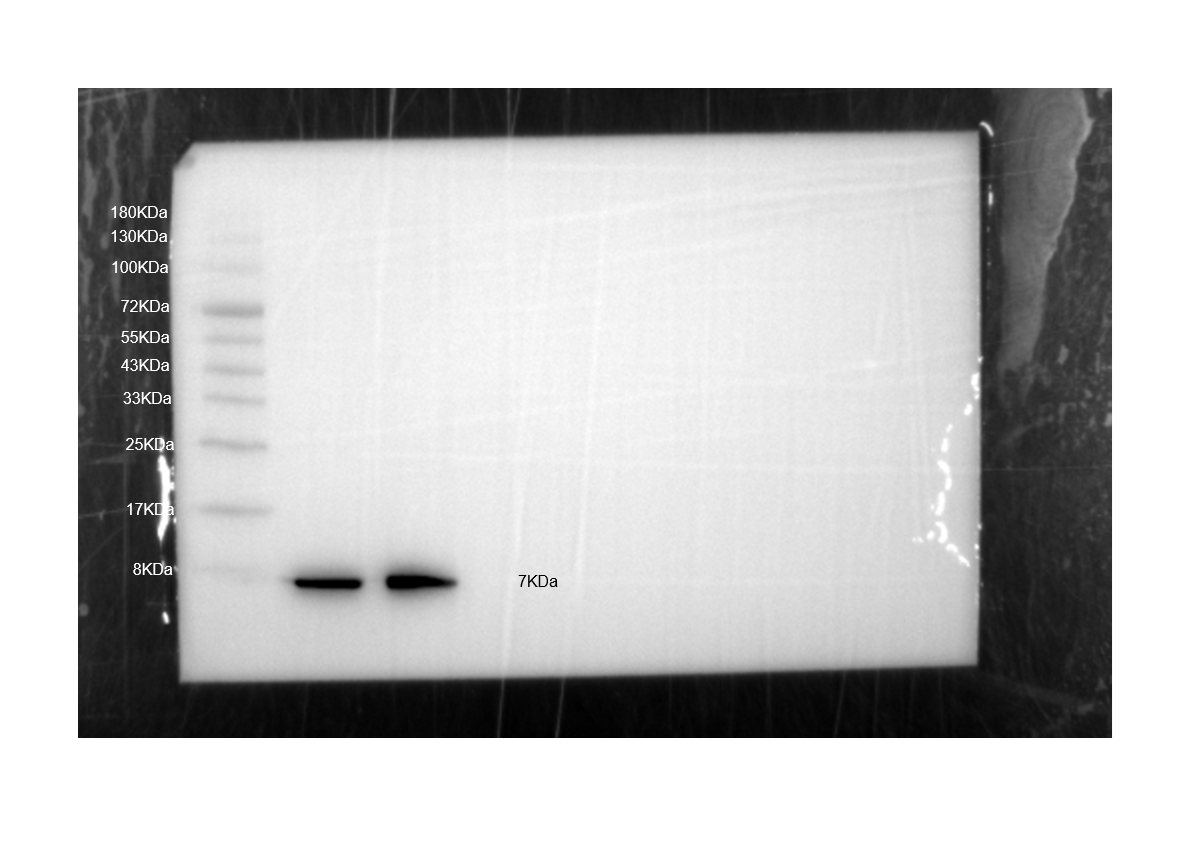


7B/MYCN


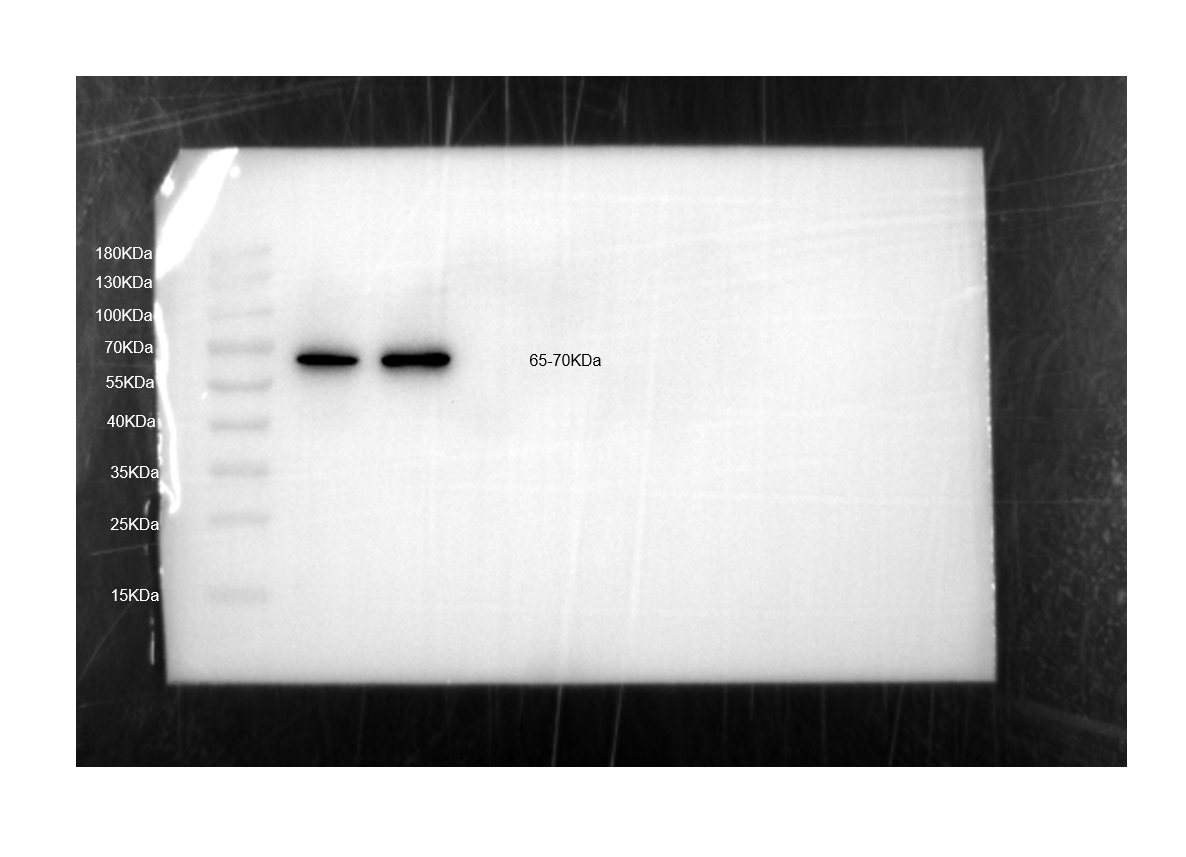


7B/NDRG1


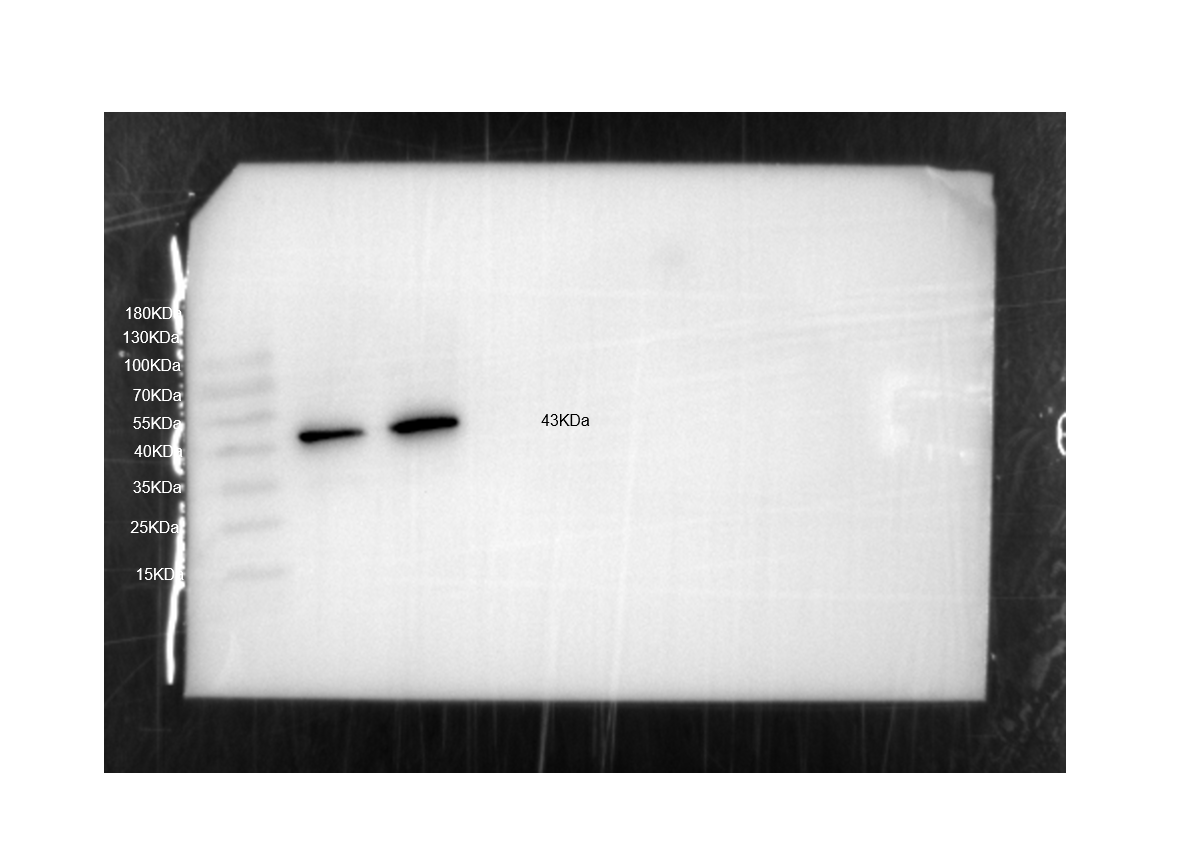


7B/PHOSPHO2


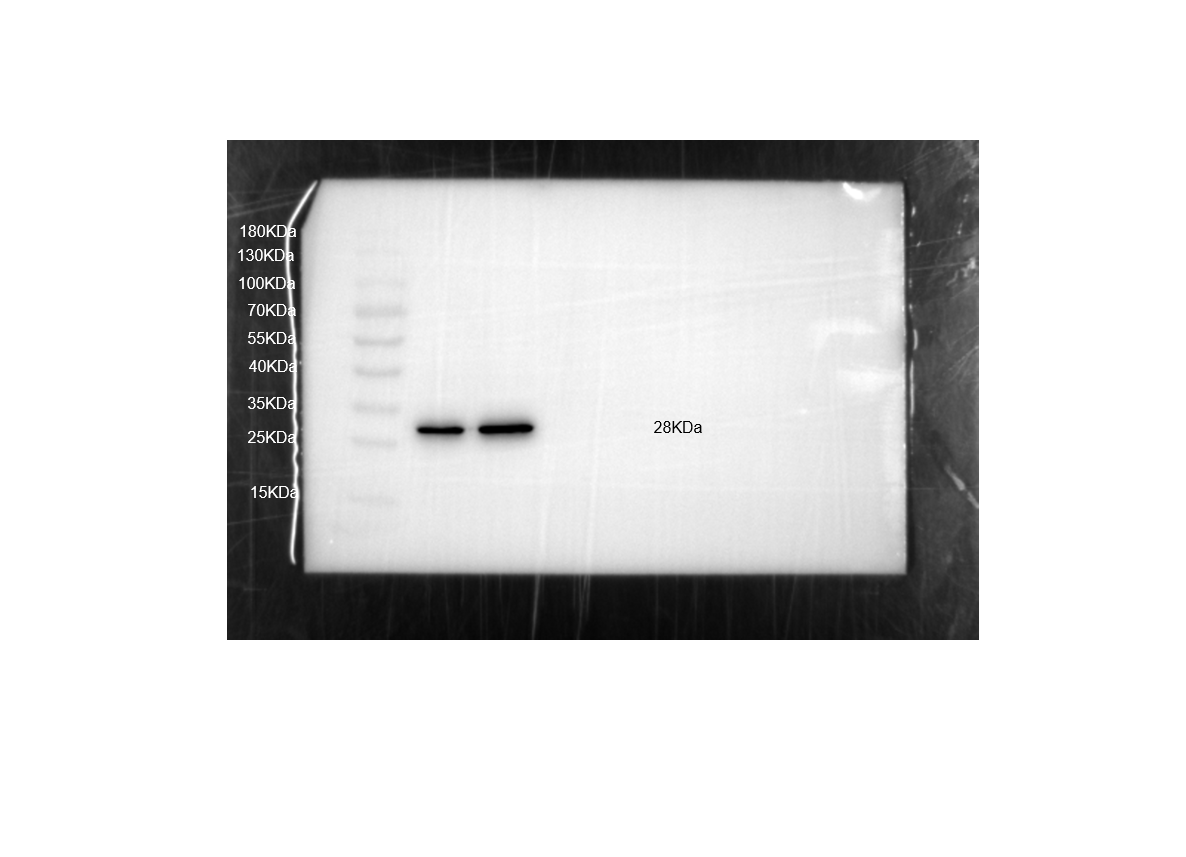


7B/SNAPC2


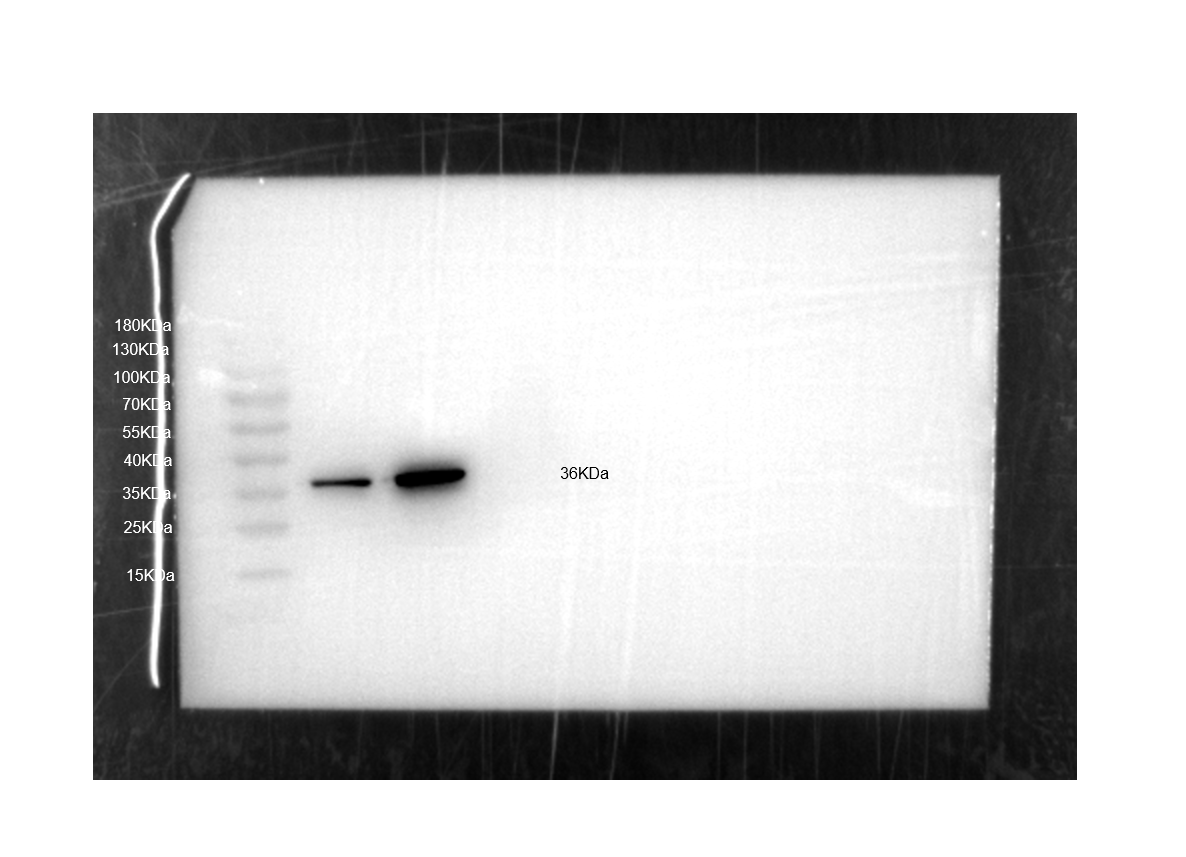


7B/SOCS2


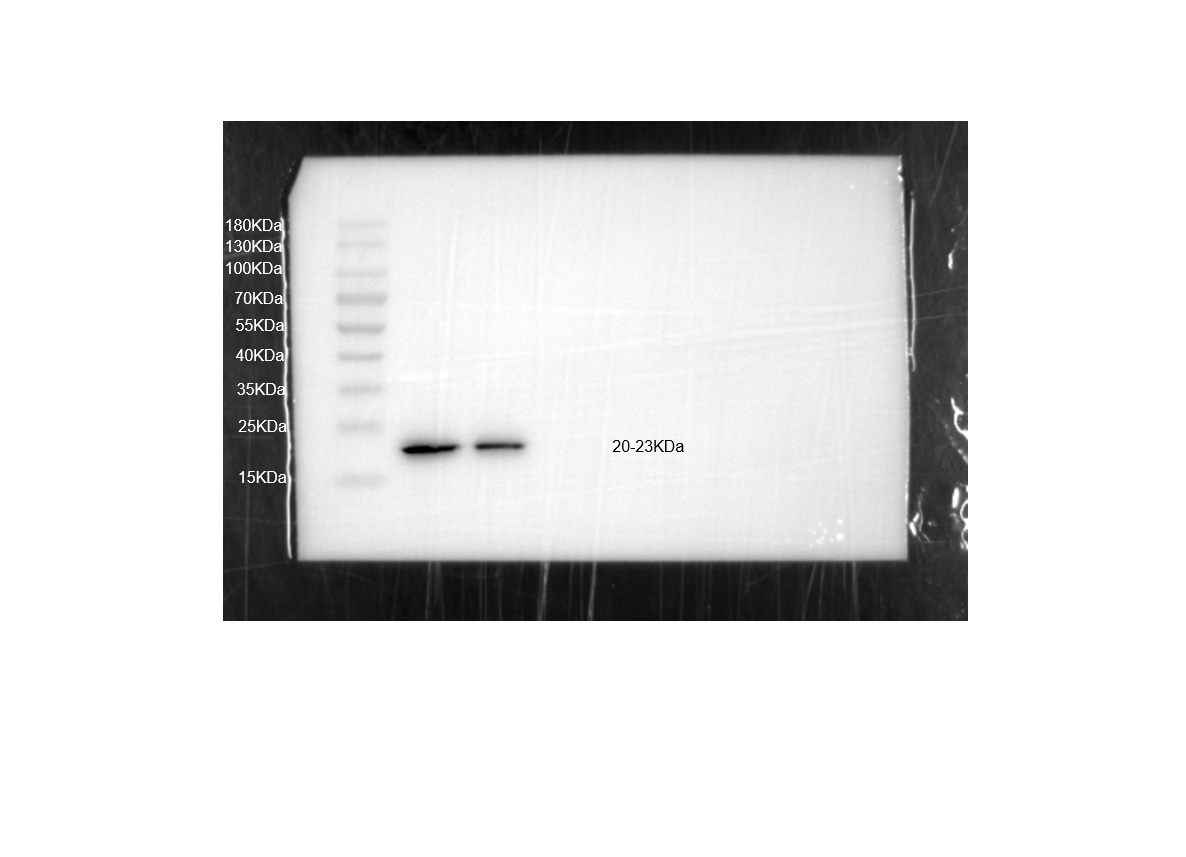


7B/TXNRD1


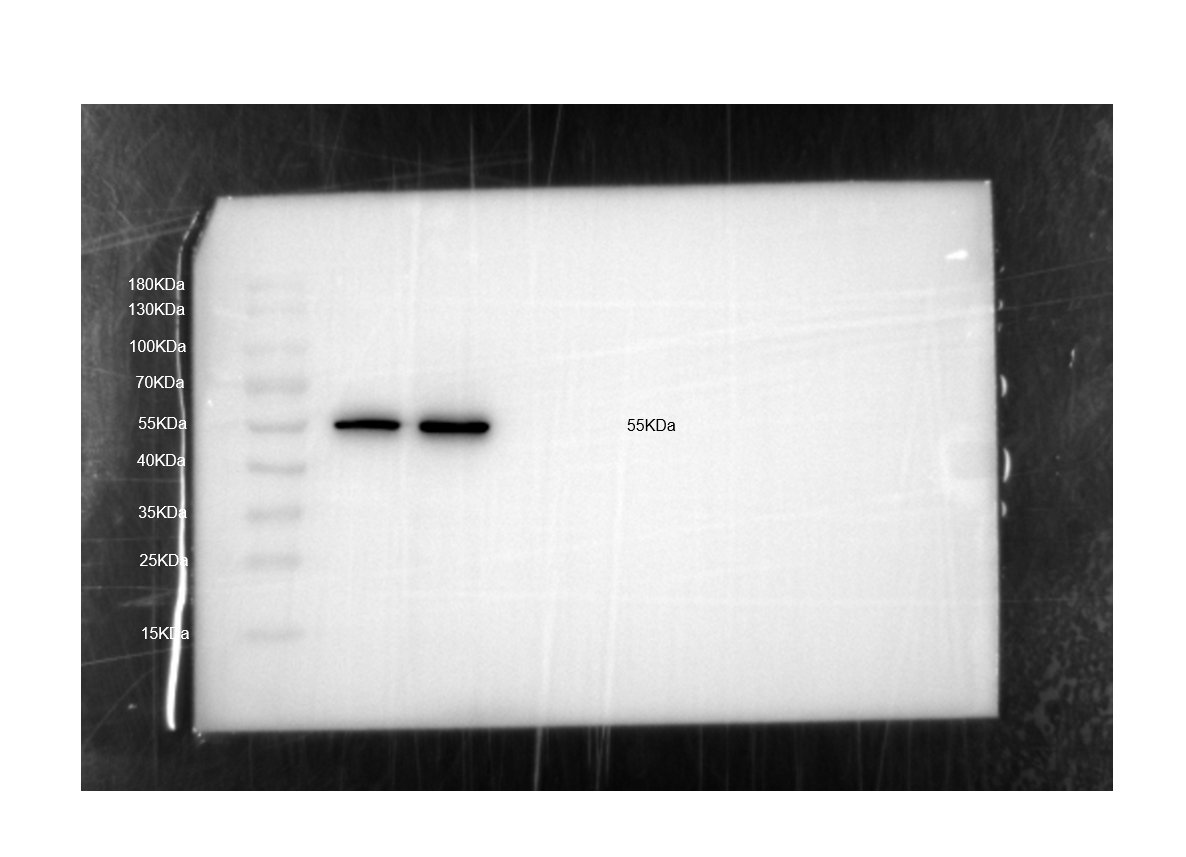


7D/GAPDH


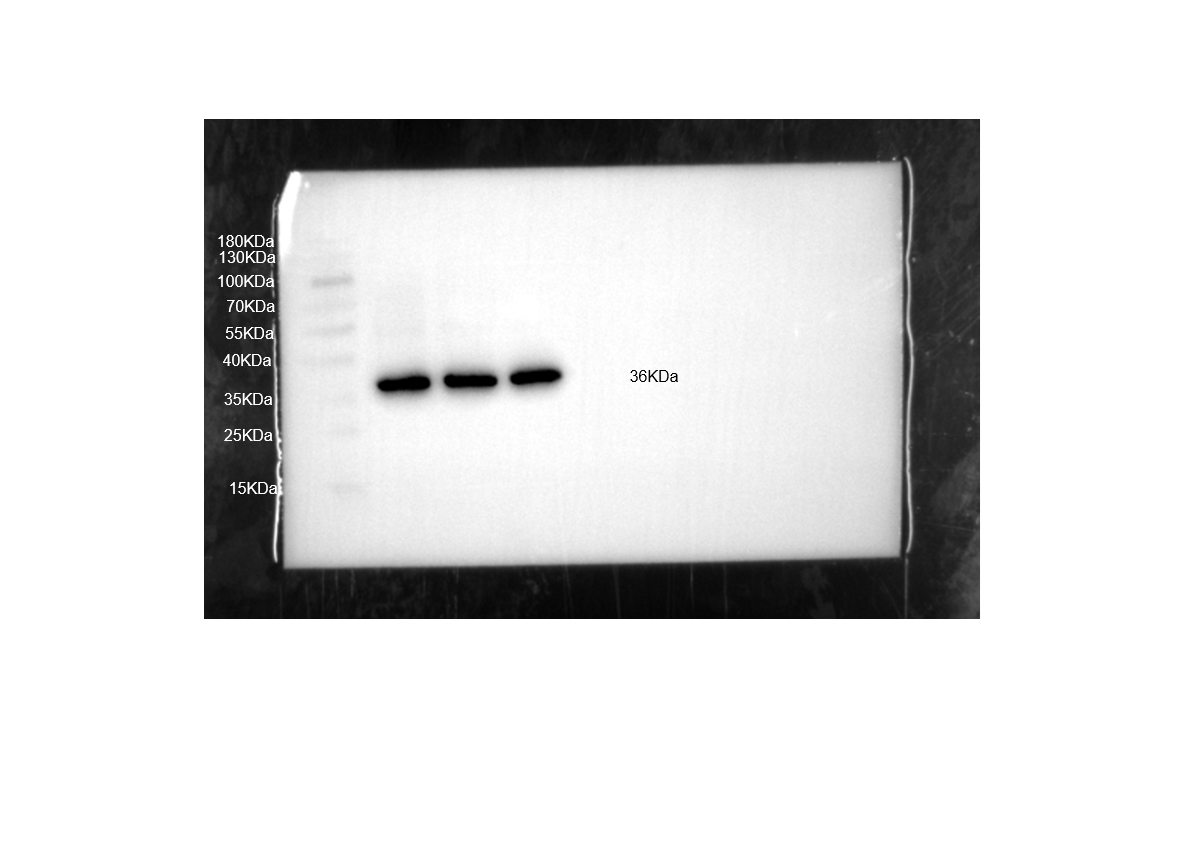


7D/SNAPC2


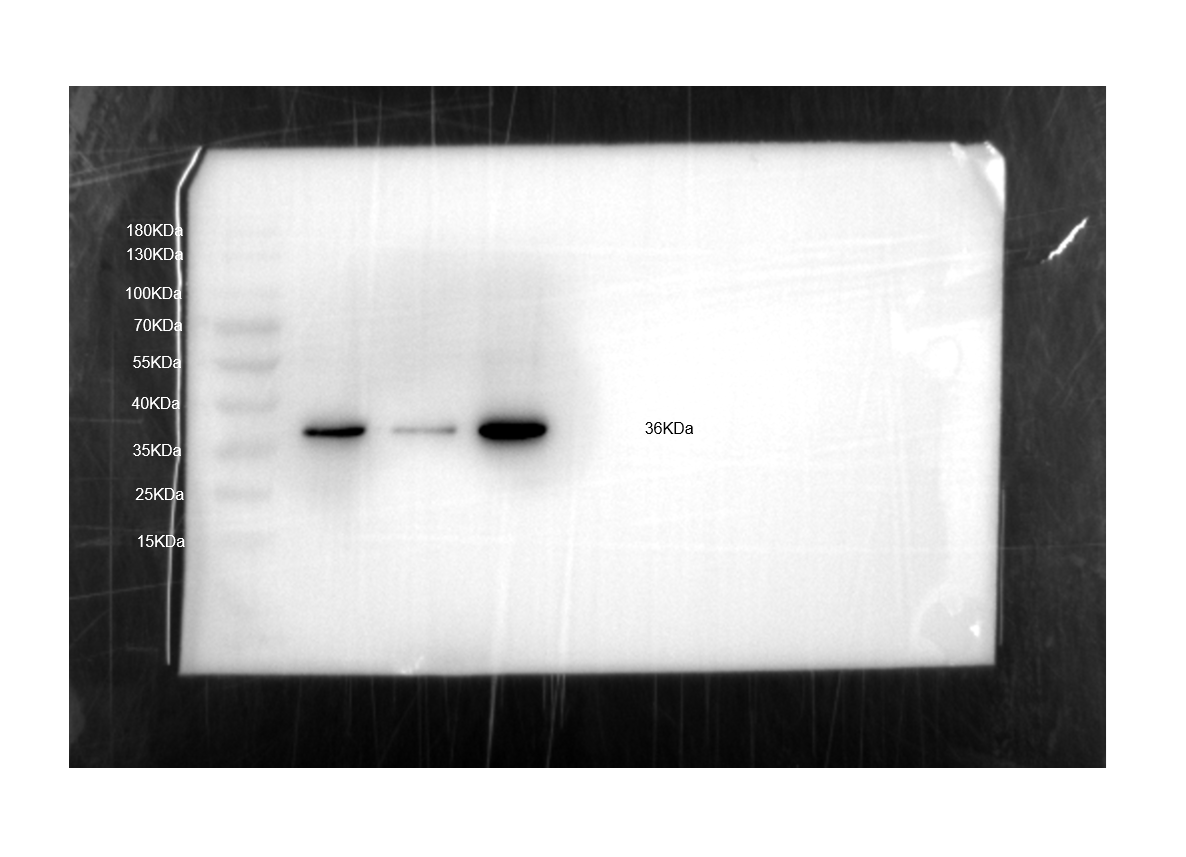

Supplement: S1 File — (DOCX) [file pone.0325610.s001.docx]
